# Assessment of knowledge and socioeconomic status of caregivers of children with malnutrition at a district hospital in Ghana

**DOI:** 10.4314/ahs.v23i1.74

**Published:** 2023-03

**Authors:** Rita Ameyaw, Emmanuel Ameyaw, Jacob K Agbenorhevi, Charles Kumi Hammond, Benard Arhin, Taiba Jibril Afaa

**Affiliations:** 1 Nursing and Midwifery Training College, P.O. Box 110, Kumasi, Ghana; 2 Department of Child Health, School of Medicine and Dentistry, Kwame Nkrumah University of Science and Technology, Kumasi, Ghana; 3 Department of Food Science and Technology, Kwame Nkrumah University of Science and Technology, Kumasi, Ghana; 4 Department of Research and Development, Komfo Anokye Teaching Hospital, Kumasi; 5 Department of Child Health, University of Ghana Medical School, University of Ghana, Accra

**Keywords:** Caregivers, knowledge, malnutrition, socioeconomic status

## Abstract

**Background:**

Malnutrition is a significant public health problem and is a major cause of morbidity and mortality in children.

**Aims:**

To assess knowledge and socioeconomic status of caregivers of children under 5 years with malnutrition at a district hospital in Ghana.

**Methods:**

Case Report forms were used to gather data in a cross-sectional study which was carried out among 189 caregivers and their children aged from zero to fifty-nine months at the Out-Patient Department clinic.

**Results:**

Most (80.95%) children had marasmus. Sixty-two point four-two percent had severe wasting, while 35.45% had mild stunting. The rest, 21.69% had moderate stunting; while only 2.12% severe stunting. Almost all caregivers (94.71%) had heard of exclusive breast feeding as a good feeding practice but only 58.20% practiced it. Most caregivers (68.26%) had no education or only up to basic level, p=0.035. The average number of children per family was 4.8 per household (SD: 1.69) with majority of them (64.55%) having 4-6 children per family and 13.23% of them had more than 7 children per family, p=0.009. More than a third (37.04%) of the caregivers earned less than a hundred Ghana cedis (GhC 100 [US$ 19] per month and 50.26% of them earned between GhC 101 (US$ 19) and GhC 500 (US$ 96) per month.

**Conclusion:**

Lower level of caregivers' education and large family size were risk factors for developing malnutrition among children.

## Introduction

Malnutrition is still a major public health problem in low-income countries including Ghana, although there have been several interventions to deal with it. Globally, it is the underlying cause of over 50% of the 10-11 million deaths among children under 5 years^1^,^2^. Severe acute malnutrition is defined as weight-for-height measurement of 70% or less below the median or three standard deviations below the mean of National Centre for Health Statistics reference values. It can also be defined as the presence of bilateral pitting edema of nutritional origin or mid-upper arm circumference of less than 110 mm in children between 1–5 years of age^1^. It is estimated that 1.4 billion people live in poverty and 25% of people are too poor to acquire food, shelter, maintain good health or educate their children^3^.

Malnutrition is a major cause of death among children under 5 years in Ghana. In Ghana, 40% of children are stunted because of poor nutrition^4^. Ghana lost an estimated 4.6 billion Ghana cedis (US$ 884.6 million) to childhood undernutrition^5^ in 2012. Poor nutrition is a major concern in Ghana with 40% of children suffering from chronic malnutrition^6^,^7^.

Malnutrition is brought about by many factors such as large family size, occupation of parents, marital status, family income, parental education, poor maternal nutritional knowledge, place of residence, gender and breastfeeding practices^3^,^8^. Socioeconomic status has impact on health seeking behavior of caregivers and nutritional status of children^3^,^9^,^10^. However, family income available to be spent on food has direct impact on child health. According to Fobil and others^10^, socioeconomic status is one of the factors that results in disparities in health of a population. In Ghana, 45% of the population is estimated to be completely poor with earnings of less than US$ 1.00 (GhC 5.20) per day^10^,^11^. Moreover, most people lack access to food, clean drinking water, toilet facilities and quality health care^6^,^10^.

Children from poor households are at greater risk of chronic undernutrition compared to those from rich households^3^,^8^,^11^. According to the 2014 Ghana Demographic Health Survey (GDHS), 22.97% of children from poorer homes suffered from malnutrition compared to 17.13% from wealthier homes^13^.

A major limitation to proper child care practices is low maternal education because maternal education equips mothers with knowledge that positively influences child nutrition and overall well-being^2^,^14^. Malnutrition related morbidity and mortality can be reduced or even prevented, when maternal knowledge on nutrition, sanitation and common disease prevention strategies are improved^2^,^11^,^13^. According to the 2014 GDHS^13^, mothers with secondary education had the least number of children (11.33%) with malnutrition compared to mothers with no education who had 26.94% malnourished children. This shows that mothers who are educated are more cautious about health of their children and take good care of them including adequate feeding.

This study was done to assess knowledge and socioeconomic status of caregivers of children under 5 years with malnutrition in Ghana.

## Methods

This was a cross sectional study which was conducted at the outpatient clinic of Maternal and Child Health Hospital (MCHH) located within Kumasi Metropolis. MCHH is a district hospital of 65 bed capacity with the children ward having 33 beds. It is a recognized center for nutritional rehabilitation for children with all types of malnutrition.

The study was conducted from 7^th^ January, 2019 to 30^th^ April, 2019. The caregivers of children with malnutrition aged between 0 and 59 months who reported to MCHH within the study period were recruited. The recruitment was conducted at the OPD clinic of the Nutritional Rehabilitation Center of MCHH.

Within the study period, 200 children were referred from various health facilities to MCHH with suspected diagnosis of malnutrition. Five of them were found not to be malnourished and so they were excluded. Six of the caregivers refused to give their consent to be included in the study. So, a total of 189 participants were recruited and interviewed after they gave informed consent. Participation was entirely voluntary and participants could withdraw from the study at any time without any consequences.

Caregivers' knowledge in child nutrition and their socioeconomic status were assessed using structured questionnaire. Practical knowledge was assessed by asking caregivers about their knowledge on breastfeeding, complementary feeding and weaning practices. So, they were asked whether they had heard of exclusive breast feeding, and whether they actually practiced exclusive breast feeding. Those who responded in the affirmative, were further asked how long they had practiced exclusive breast feeding so as to determine whether they did it up to the recommended six months^6^,^13^. They were also asked about what age they introduced complementary feeds and when they weaned their children completely off breast milk. Employment, educational status and income levels were used to assess socioeconomic status of the caregivers.

The types of acute malnutrition such as kwashiorkor, marasmus and marasmic/kwashiorkor were determined using weight for height. The Mid Upper Arm Circumference (MUAC) was assessed for children aged six months to fifty-nine months. Height for age, weight for age and edema of the feet and hands were also assessed.

Length of children less than 2 years of age was taken using an infantometer (Seca 416). The child was put in a supine position on the infantometer placed on a flat surface. The head was positioned firmly against the fixed end and the knees were extended and the legs straightened with the feet fixed at right angles to the infantometer. The sliding foot positioner was adjusted and the length was recorded to the nearest 0.1cm.

Height of children older than 2 years was measured using microtoise attached to a smooth straight wall. The children were asked to stand without shoes and with their feet at right angles and their back against the wall. The headpiece was then gently lowered to touch the top of the head and height was recorded to the nearest 0.1cm.

Weight of children under five years weighing less than 23 kilogram (kg) was measured using toddlers weighing scale while those who weighed more than 23 kg had their weight measured using the adult analog weighing scale to the nearest 0.1kg. Weight was measured with the child wearing no clothing or only in under-pants.

MUAC was measured for children aged 6 to 59 months using the left arm with no clothing. The arm was bent at right angles at the elbow to the body and the mid-point of the upper arm was located between the tip of the shoulder and the elbow and was marked with ink. Shakir tape was then used to measure MUAC to the nearest millimetre (mm).

Weight for height (wasting), weight for age (underweight) and height for age (stunting) were computed using WHO standard Z score for measuring undernutrition^15^.

### Statistical Analysis

Data were entered into Excel 2016 from the CRF and then transported onto StataSE 16 (StataCorp, Texas 77845, USA), for analysis. Univariate analysis with point estimates were presented in frequencies and percentages. Fisher's Exact test was used to assess differences in the means of continuous variables and significant levels were assessed using p <0.05.

### Ethical Approval

The Committee on Human Research, Publications and Ethics (CHRPE) of Kwame Nkrumah University of Science and Technology (KNUST) gave ethical approval for the study protocol.

## Results

### Demographic characteristics of Caregivers of children under 5 with malnutrition

From Table 1, a total of one hundred and eighty-nine caregivers whose children were diagnosed with malnutrition were recruited. Most (91.53%) were biological mothers. The median age of caregiver was 30 years (IQR: 35-25). The youngest caregiver was 17 years old while the oldest was 65. Most caregivers (83.07%) were Christians, 16.40% were Muslims and one (0.53%) was of Traditional African Religion faith. Most caregivers (67.20%) were of the Akan tribe. More than two thirds of the caregivers (67.77%) were married; 44.44% were separated and 21.16% were cohabiting. The average number of children per family was 4.8 (SD: 1.69) with most (64.55%) having 4-6 children per family. Only 13.23% of them had more than 7 children per family. Up to 68.26% of the caregivers had no formal education or only up to basic level, only 9.52% had vocational, teacher training college or university education. Most of the caregivers were either unemployed (22.22%) or worked in the informal sector (68.25%). With regard to income levels, 37.04% of the caregivers earned less than 100 Ghana cedis (US$ 19.23) per month while 50.26% earned between GhC 101 (US$ 19.42) to GhC 500 (US$ 96.15) per month.

**Table 1:** Demographic characteristics of Caregivers of children under 5 with malnutrition

Variables	Categories	Frequency (%)
**Caregivers**		
	Mother	173 (91.53)
	Grandmother	10 (5.29)
	Father	3 (1.59)
	Aunt	3 (1.59)
**Age of caregivers (years)**		
	16–25	48 (25.40)
	26–35	99 (52.38)
	36–45	29 (15.34)
	46–55	10 (5.29)
	56–65	3 (1.99)
**Religion of caregivers**		
	Christianity	157 (83.07)
	Islam	31 (16.40)
	Traditional religion	1 (0.53)
**Tribe of caregivers**		
	Akan	127 (67.20)
	Ewe	2 (1.06)
	Ga	2 (1.06)
	Dagomba	3 (1.59)
	Others	55 (29.10)
**Marital status of caregivers**		
	Married	128 (67.72)
	Cohabiting	40 (21.16)
	Divorced	10 (5.82)
	Separated	11 (5.82)
**Family size**		
		42 (22.22)
		121 (64.02)
		26 (13.76)
**Educational level**		
	No Education	30 (15.87)
	Primary School	16 (8.47)
	Junior High School	83 (43.92)
	Senior High School	42 (22.22)
	Training College	6 (3.17)
	Vocational School	5 (2.65)
	University	7 (3.70)
**Occupation of caregivers**		
	Formal	18 (9.52)
	Informal	129 (68.25)
	Unemployed	42 (22.22)
**Income levels (GHC)**		
	Less than 100	70 (37.04)
	Between 100 to 500	95 (50.26)
	Between 501 to 1000	11 (5.82)
	1001 to 5000	11 (5.82)
	5001 to 10000	2 (1.06)

### Demographic characteristics of malnourished children

From Table 2, the median age of a child with diagnosis of malnutrition recruited was 14 months (IQR: 9-20). The youngest child with malnutrition was 2 months and the oldest was 59 months old. Most (88.88%) children were up to 24 months of age. Males were 103 (55.5%) of the malnourished children. Most (84.13%) children had been registered under the National Health Insurance Scheme (NHIS), while 11.64% had not been registered and 4.23% had been registered but their registration status had expired.

**Table 2:** Demographic characteristics of malnourished children

Variable	Category	Frequency (n%)
Age of children (months).		
	0–12	84 (44.44)
	13–24	84 (44.44)
	25–36	15 (7.94)
	37–48	3 (1.59)
	49–59	3 (1.59)
Sex of child.		
	Male	103 (55.5)
	Female	86 (45.5)
National Health		
Insurance Scheme.		
	Access	159 (84.13)
	No Access	22 (11.64)
	Expired	8 (4.23)

### Nutritional status of children with malnutrition

As seen in Table 3, most children (80.95%) had marasmus; 11.64% marasmic/kwashiorkor and 7.41% had kwashiorkor. Wasting was assessed using weight for height and 62.42%, found to be severe 23.70% moderate and 13.87% mild. With regard to underweight 32.28% was diagnosed as mild and 4.76% as severe while for stunting 2.12% was severe; 21.69 moderate and 35.45% mild. Assessment using MUAC, 90.29% of the children had severe wasting while 9.71% had moderate wasting. On clinical examination, 80.95% of the malnourished children had weight loss, 6.88% had generalized edema or edema of both feet whereas 12.17% had wasting with some edema.

**Table 3:** Nutritional status of children with malnutrition

Variables	Type	Frequency (n%)
**Type of malnutrition**		
	Kwashiokor	14 (7.41)
	Marasmus	153 (80.95)
	Marasmic Kwashiokor	22 (11.64)

**Weight for height/length (wasting)**	Severe	108 (62.42)
	Moderate	41 (23.70)
	Mild	24 (13.87)

**Weight for age (underweight)**	Severe	9 (4.76)
	Moderate	95 (50.26)
	Mild	61 (32.28)
	Normal	24 (12.70)

**Height for age (stunting)**		
	Severe	4 (2.12)
	Moderate	41 (21.69)
	Mild	67 (35.45)
	Normal	77 (40.74)

**Mid upper arm circumference (cm)**		
	Severe wasting	158 (90.29)
	Moderate wasting	17 (9.71)

**Clinical features**		
	Edema of feet/body	13 (6.88)
	Weight loss	153 (80.95)
	Weight loss/Edema	23 (12.17)

### Comparison of caregivers' knowledge and breastfeeding

From Table 4, from multinomial logistic regression analysis using kwashiorkor as base line, introduction of water at age less than three months of age was significant for development of marasmic kwashiorkor, p=0.037 whereas giving water rather at birth was significant in developing marasmus, p=0.047. introduction of complementary feeds from birth to six months of age did not pose as risk for developing malnutrition, p=0.683.

**Table 4:** Comparison of caregivers' knowledge and breastfeeding

Type of Malnutrition		RRR (Base outcome)	Std.Err.	z	P>z	[95% Conf. Interval]	
**Kwashiokor**	**Age that water was** **introduced**						
	At birth	0.2356655	0.2573885	- 1.32	0.186	0.0277098	2.004282
	Between three and three and six months	0.9917092	1.085148	- 0.01	0.994	0.1161401	8.468109
	Less than three months	0.0527037	0.0744875	- 2.08	0.037	0.0033023	0.8411361
**Marasmic** **Kwashiokor**	**Age that** **complementary** **feeds added**						
	At six months	0.6300246	0.7057976	- 0.41	0.68	0.0701103	5.661519
	Between three and six months	0.4162183	0.5144478	- 0.71	0.478	0.0369175	4.692567
	Less than three Months	3.208806	4.959977	0.75	0.451	0.1550986	66.38639
	**Family size**	1.785586	0.6542579	1.58	0.114	0.8707453	3.661594
	**Constant**	0.4734558	0.8664584	- 0.41	0.683	0.0131075	17.10173

**Marasmus**	**Age that water was** **introduced**						
	At birth	0.1613491	0.1481556	- 1.99	0.047	0.0266789	0.9758108
	Between three and three and six months	0.6538235	0.6320757	- 0.44	0.66	0.0983042	4.348594
	Less than three Months	0.2336925	0.2138742	- 1.59	0.112	0.0388713	1.404947
	**Age that** **complementary** **feeds added**						
	At six months	0.5292923	0.4943427	- 0.68	0.496	0.0848593	3.30135
	Between three and six months	0.9529787	0.9373864	- 0.05	0.961	0.1386145	6.551756
	Less than three Months	3.511185	4.620718	0.95	0.340	0.2662399	46.3057
	**Family size**	2.363017	0.7821836	2.6	0.009	1.235123	4.520886
	**Constant**	0.815253	1.309946	- 0.13	0.899	0.0349611	19.01079

Note Multinomial logistic regression: constant estimates baseline relative risk for each outcome

### Comparison of socio-economic status of caregivers with type of malnutrition

From table 5, from multinomial logistic regression analysis using kwashiorkor as base line, caregivers' age, occupational status of caregivers, educational status and income levels were not significant in developing marasmic kwashiorkor in the children. Caregivers' age, occupational status of caregivers and income levels were not significant in developing marasmus whereas vocational education was significant for development of marasmus children, p=0.035. Family size was significant for developing marasmus, p=0.00

**Table 5:** Comparison of socio-economic status of caregivers with type of malnutrition

Type of Malnutrition		RRR	Std. Err.	z	P>z	[95% Conf. Interval]	

**Kwashiokor**	(base outcome)						
**Marasmic** **Kwashiokor**	**Caregiver Age**						
	25–35	0.5914591	0.6105205	-0.51	0.611	0.0782152	4.472585
	36–50	0.8505281	1.042121	-0.13	0.895	0.0770447	9.389335
	>50	0.062274	313.5943	0	1	0	.
	**Occupation**						
	Informal	1.21E-08	0.00008	0	0.998	0	.
	Unemployed	2.17E-08	0.0001438	0	0.998	0	.
	**Education** **Status**						
	No education	2.145941	2.745743	0.6	0.551	0.1747839	26.34719
	Primary	1.07E-08	0.0000439	0	0.996	0	.
	SHS	0.256777	0.2808836	-1.24	0.214	0.0300914	2.191138
	Training	COLLEGE	1.38E+08	#####	0	0.999	0
	University	0.7076452	7169.968	0	1	0	.
	Vocational	1.58E-11	2.45E-06	0	1	0	.
	**Income**						
	¢5001–10000	1.802856	26634.13	0	1	0	
	¢101–500	5.75512	85022.13	0	1	0	
	<¢100	3.148075	46507.46	0	1	0	
	**Constant**	4.89E+07	7.91E+11	0	0.999	0	.
**Marasmus**							
	**Caregiver age**						
	25–35	1.777017	1.522435	0.67	0.502	0.3314639	9.526799
	36–50	2.740309	2.804383	0.99	0.325	0.3687187	20.36591
	>50	7342985	1.15E+10	0.01	0.992	0	.
	**Occupation**						
	Informal	6.81E-08	0.0004506	0	0.998	0	.
	Unemployed	5.37E-08	0.000355	0	0.998	0	.
	**Education** **status**						
	No education	1.393108	1.593379	0.29	0.772	0.1480524	13.10853
	Primary	0.4903155	0.4611009	- 0.76	0.449	0.0776232	3.097133
	SHS	0.6480416	0.493824	- 0.57	0.569	0.1455345	2.885625
	Training college	4.59E+07	8.05E+11	0	0.999	0	.
	University	2.986236	30256.99	0	1	0	.
	Vocational	0.0832585	0.0978996	- 2.11	0.035	0.0083089	0.8342857
	**Income**						
	¢5001–10000	1.79E-08	0.0002356	0	0.999	0	
	¢101–500	6.13E-08	0.0008091	0	0.999	0	
	<¢100	5.64E-08	0.0007439	0	0.999	0	
	**Constant**	2.22E+15	3.27E+19	0	0.998	0	

Note Multinomial logistic regression: constant estimates baseline relative risk for each outcome

## Discussion

More males were affected with malnutrition compared to females. This is in conformity with GDHS report^13^ in 2014 which indicated that male children are more likely to be malnourished compared to their female counterparts. This is probably due to the differences in biological composition between the female and the male child18 and also preferential care for girls due to daughter preference^19^ among certain tribes in Ghana.

Malnourished children identified in our study fell into three categories thus marasmus, marasmic kwashiorkor and kwashiorkor. This is similar to the three types of protein energy malnutrition described by Muller and Krawinkel^20^. Furthermore, the study revealed that children below 24 months were the most affected. This is consistent with the findings of GDHS^13^ in 2014, where more children below 24 months were affected with malnutrition. This could be due to the fact that in Ghana significant numbers of women give birth at shorter interval of less than 24 months^16^. Children from such mothers suffer malnutrition because nutritional attention shifts to the newborn babies.

Our study revealed that introducing water at age less than three months was a risk factor for developing maramic kwashiorkor while giving water at birth pose risk for developing marasmus in children. For good infant nutritional practices, WHO and United Nations Children's Fund (UNICEF) recommend six months exclusive breastfeeding as well as introduction of complementary feeding from 6 to 24 months^7^,^14^ meanwhile a good proportion of the caregivers breastfed their children for less than six months. More than a half of the caregivers added complementary feeds to the breast milk before the children attained 6 months of age which was also consistent with the findings of GDHS^13^ in 2014, where 12% of children between 2 to 3 months and 34% of children between 4 to 5 months were introduced to complementary foods. However, our study revealed that introduction of complementary feeds within the first 6 months did not contribute to development of malnutrition among children. This probably could be due to our sample size.

A higher proportion of the participants had no education or only up to basic level and this more probably contributed to their children developing malnutrition. This is consistent with the study conducted by Saaka^2^ in Northern Ghana where he found that most of the mothers of children with malnutrition had no formal education. It is also in agreement with studies by Amsalu et al9 and Hong^11^ which indicated that mother's formal education improves on hygiene and good feeding practices of their children.

Most caregivers were unemployed and up to 87.30% earned less than GhC 500 (US$ 96.15) per month. This finding supports what Hong^11^ found in Ghana that 45% of the Ghanaian population is poor and earn less than US $ 1 (GhC 5.20) per day^11^. It is also similar to the findings of GDHS^13^ in 2014 where most malnourished children came from poor homes. None the less, our study revealed no association between the occupation of caregivers and their income level and children developing malnutrition probably because the study population was homogenous. Most participants had large family size of more than 4 children per family, with some families having as many as 10 children, and this might have contributed to some of the children developing malnutrition in the form of marasmus. This was similar to findings by Amsalu and Tigabu^9^ who observed that large family size was a risk factor for developing severe acute malnutrition. Because the more children there are in a family the more income the family would need for their upkeep including feeding.

## Limitation

The study was conducted at only Maternal and Child Health Hospital, Kumasi and with a little sample size. The study could be replicated in many centers with bigger sample size.

## Conclusion

Caregivers with no education or low educational status and large family size are risk factors for childhood malnutrition. Almost all the caregivers knew of the importance of exclusive breastfeeding for the first six months but most of them did not practice it. Early introduction of complementary feeding was common among caregivers contrary to WHO and UNICEF recommendation of six months exclusive breastfeeding.

With the free senior high school and free school feeding policies in Ghana, many more girls are being admitted and retained in school than before. This will lead to more women with higher educational status which will in the long-term result in improvement in nutrition among children in Ghana. With active implementation of other policies such as planting for food and jobs and one district one factory food is being produced in abundance and jobs being created for the people of Ghana. It is, therefore, hoped that with continuing education on childhood nutrition at the ante natal and post natal clinics, malnutrition among children will reduce significantly.
